# Arterite de Células Gigantes com Envolvimento Aórtico Cursando com Síndrome Cardiovocal (Síndrome de Ortner)

**DOI:** 10.36660/abc.20180427

**Published:** 2020-05-11

**Authors:** Edgar Stroppa Lamas, Ricardo Luiz José Rogoni Bononi, Paulo Augusto Cotta de Ávila Reis

**Affiliations:** Hospital 13 de Maio Departamento de Cardiologia SorrisoMT Brasil Hospital 13 de Maio , Departamento de Cardiologia , Sorriso , MT – Brasil

**Keywords:** Arterite de Células Gigantes, Aneurisma de Aorta Torácica/fisiopatologia, Síndrome Cardiovocal, Traumatismos do Nervo Laringeo Recorrente, Síndrome de Ortner

## Introdução

Arterite de células gigantes ou arterite temporal é a arterite de grandes vasos mais comum em países ocidentais, acometendo geralmente pacientes acima de 50 anos. ^1^ Na maioria dos casos sintomas cranianos estão presentes, no entanto, manifestação exclusivamente extracranianas pode ocorrer em até 22% dos casos. ^2^

O envolvimento aórtico e de outros grandes vasos (carótidas, subclávias) é frequente neste contexto. Observa-se acometimento da adventícia da artéria principalmente por ativação de células dendríticas gerando processo inflamatório granulomatoso em focos múltiplos com intensa presença linfocítica. A ocorrência de aneurismas de aorta torácica é reportado em cerca de 20% dos pacientes. ^3^

A síndrome cardiovocal também conhecida com Síndrome de Ortner caracteriza-se pelo acometimento não maligno do nervo laríngeo recorrente secundário a causas cardiovasculares principalmente patologias que levam aumento do átrio esquerdo e aneurisma da aorta torácica. Trata-se de condição rara que leva a rouquidão por compressão do referido nervo. ^4^

Neste relato descrevemos um caso incomum de uma paciente com arterite de células gigantes com manifestações extracranianas por acometimento da aorta torácica culminado com síndrome cardiovocal. Associação esta rara, nunca descrita na literatura brasileira.

## Relato de caso

Paciente de 65 anos atendida em ambulatório de cardiologia com queixa de febre iniciada há 30 dias, hiporexia e perda ponderal. Nos últimos 10 dias iniciou quadro de dor torácica a esquerda contínua sem caráter anginoso, motivo pelo qual foi encaminhada a consulta cardiológica após investigação inicial com infectologista. Antecedentes patológicos incluíam hipertensão arterial sistêmica de longa data e passado de tabagismo (carga tabágica de 20 anos-maço).

Ao exame físico paciente apresentava regular estado geral e hipocorada. Ausculta cardíaca e pulmonar sem anormalidades. Sopro carotídeo à direita. Pulso radial e braquial esquerdo ligeiramente diminuído em relação ao direito. Pressão arterial aferida mostrava 140/80 mmHg e 120x70 mmHg no membro superior direito e esquerdo respectivamente.

Exames laboratoriais evidenciam anemia normocrômica e normocítica (Hb: 10,6 g/dL), velocidade de hemossedimentação (VHS) de 115 mm/1hora) e proteína c-reativa (PCR) 48 mg/L. Demais exames laboratoriais sem alterações dignas de nota. Solicitado doppler arterial de vasos cervicais que mostrou aumento de espessura do complexo médio intimal e placa obstrutiva de 70% na artéria carótida interna direta. Observa-se também fluxo retrógado da artéria vertebral esquerda para artéria subclávia esquerda (síndrome do roubo da artéria subclávia tipo III).

**Figura 1:**
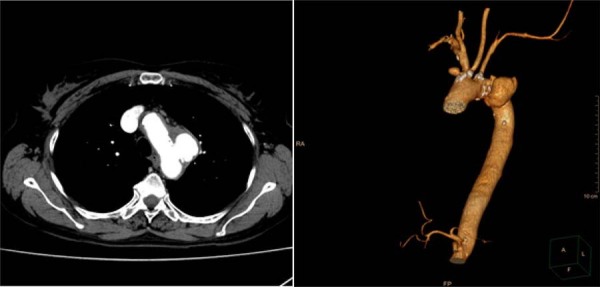
- Angiotomografia da aorta torácica. A) Dilatação aneurismática sacular após a emergência da subclávia esquerda, parcialmente trombosada, medindo 4,2 cm de comprimento e 2,4 x 2,8 cm nos maiores diâmetros. B) Imagem obtida após reconstrução.

Diante dos achados de comprometimento carotídeo e da subclávia em paciente com sintomas constitucionais, com provas de atividades inflamatórias elevadas e dor torácica foi solicitada angiotomografia de aorta torácica. Exame evidenciou dilatação aneurismática da aorta torácica com importante espessamento parietal logo após a emergência da artéria subclávia esquerda com diâmetro de 24x28 mm ( Figura 1 ).

Baseado nos critérios diagnósticos do *American College of Rheumatology* 1990 ^1^ foi aventada hipótese de artrite de células gigantes na forma extracraniana com comprometimento aórtico. Foi iniciada corticoterpaia com prednisona 60 mg/dia.

**Figura 2:**
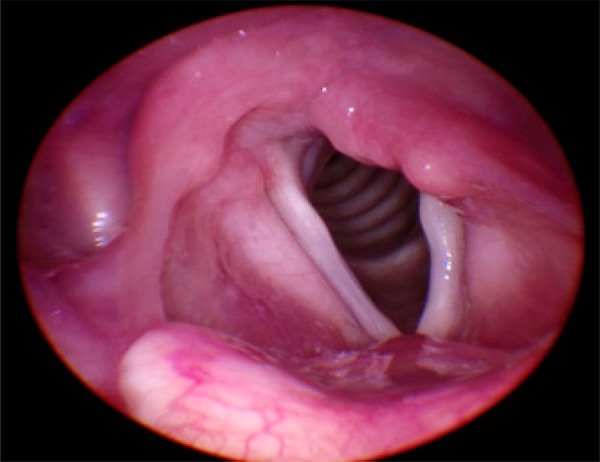
- Imagem obtida por videolaringoscopia evidencia assimetria das cordas vocais, com sinais da paralisa da corda vocal esquerda.

Nos dias subsequentes a paciente evolui com rouquidão persistente. Solicitada videolaringoscopia que evidencia paralisa da corda vocal esquerda ( Figura 2 ). Devida à correlação anatômica do aneurisma com nervo laríngeo recorrente esquerdo foi estabelecido o diagnóstico de síndrome cardiovocal (ou síndrome de Ortner).

Após corticoterapia paciente apresentou melhora significativa dos sintomas constitucionais e da dor torácica. Marcadores inflamatórios após duas semanas de tratamento apresentaram queda importante (VHS: 20 mm/1hora e PCR: 1,0g/L) e houve melhora da anemia (Hb: 12,4 g/dL). No seguimento de 30 dias a paciente mantém-se assintomática com melhora discreta da rouquidão após início de fonoterapia.

## Discussão

A arterite de células gigantes mesmo sendo uma condição com prevalência não desprezível em algumas situações pode apresentar difícil diagnóstico, principalmente quando sintomas cranianos como cefaleia temporal estão ausentes. No entanto diante de perfil epidemiológico apropriado, associado a sintomas constitucionais sem explicação evidente e indícios de acometimento de grandes vasos seu diagnóstico deve ser aventado.

Trata de condição mais frequente no sexo feminino na proporção de 4:1. História de tabagismo, como no nosso caso, aumento em creca de 6 vezes o risco de desenvolvê-la. ^5^ Os critérios diagnósticos foram estabelecidos em 1990 pelo *American College of Rheumatology* . A biópsia de artéria temporal evidenciando padrão inflamatório granulomatoso faz parte dos critérios, no entanto, carece de sensibilidade adequada podendo ser evitada no contexto apropriado na presença de quadro clínico sugestivo e com exames de imagens compatíveis com acometimento de grandes vasos. ^6^

A presença de VHS elevada (geralmente acima de 55 mm/1hora) é dado laboratorial inespecífico, porém muito frequente. Assim com a resposta rápida e eficaz, a corticoterapia fortalece sua possibilidade diagnóstica. O acometimento aórtico não é achado infrequente e seu reconhecimento precoce bem como respectivo tratamento é fundamental para minimizar complicações agudas e crônicas. ^7^

A síndrome de Ortner ou síndrome cardiovocal, descrita pela primeira vez em 1897, trata-se de situação rara que se caracteriza pela compressão do nervo laríngeo recorrente por condições cardiovasculares levado a rouquidão, disfagia e disfonia. ^8^ Sua associação com arterite de células gigantes é extremamente rara e infrequente como apresentado no nosso caso. ^9^

Este caso apresenta uma associação incomum cujo reconhecimento é fundamental para correto manejo e terapêutica apropriada no intuito de minimizar complicações adjacentes da arterite de células gigantes.
