# Supplementary material for: Arterite de Células Gigantes com Envolvimento Aórtico Cursando com Síndrome Cardiovocal (Síndrome de Ortner)
Source: Arq Bras Cardiol. 2020 May 11;114(4 Suppl 1):16–8. [Article in Portuguese] doi: 10.36660/abc.20180427 (PMC8149116; doi:10.36660/abc.20180427)
Supplement: Translation

# Translation: Giant Cell Arteritis with Aortic Involvement Leading to Cardio Vocal Syndrome (Ortner’s Syndrome)

**Authors:** Edgar Stroppa Lamas, Ricardo Luiz José Rogoni Bononi, Paulo Augusto Cotta de Ávila Reis

**Affiliations:** Brazil Hospital 13 de Maio, Departamento de Cardiologia, Sorriso, MT – Brazil

**Keywords:** Giant Cells Arteritis, Aortic Aneurysm, Thoracic/physiopathology, Vocal Cord Syndrome, Recurrent Laryngeal Nerve, Ortner’s Syndrome

## Introduction

Giant cell arteritis or temporal arteritis is the most common large-vessel arteritis in Western countries, generally affecting patients over 50 years of age.^1^In most cases cranial symptoms are present, however, exclusively extracranial manifestations can occur in up to 22% of cases.^2^

The involvement of aortic and other large vessels (carotid, subclavian) is frequent in this context. The involvement of the adventitia of the artery is observed mainly by activation of dendritic cells generating a granulomatous inflammatory process in multiple foci with intense lymphocytic presence. The occurrence of thoracic aortic aneurysms is reported in about 20% of patients.^3^

The cardiovocal syndrome also known as Ortner’s Syndrome is characterized by non-malignant involvement of the recurrent laryngeal nerve secondary to cardiovascular causes, mainly pathologies that lead to enlargement of the left atrium and thoracic aortic aneurysm. This is a rare condition that leads to hoarseness by compression of the aforementioned nerve.^4^

In this report, we describe an unusual case of a patient with giant-cell arteritis with extracranial manifestations due to involvement of the thoracic aorta culminating with cardiovocal syndrome. This association is rare, never described in Brazilian literature.

## Case Report

At 65 years old female patient seen in outpatient cardiology with a complaint of fever started 30 days ago, hyporexia and weight loss. In the last 10 days, she started with continuous left thoracic pain without angina patterns, which is why she was referred for a cardiological appointment after an initial investigation with an infectologist. Pathological antecedents included long-standing systemic arterial hypertension and smoking history (20-year smoking load).

At physical examination, the patient presented regular general condition and skin pallor. Cardiac and pulmonary auscultation without abnormalities. Right carotid bruit. Left radial and brachial pulses slightly decreased in relation to the right. Blood pressure measured was 140/80 mmHg and 120/70 mmHg in the right and left upper limbs, respectively.

Laboratory tests showed normochromic and normocytic anemia (Hb:10.6 g/dl), Erythrocyte sedimentation rate (ESR) of 115 mm/1 hour, and C-reactive protein (CRP) of 48mg/L. Other laboratory tests with no significant changes. Arterial Doppler of cervical vessels was requested and showed increased thickness of the intima-medial complex and 70% obstructive plaque in the direct internal carotid artery. Retrograde flow from the left vertebral artery to the left subclavian artery (type III subclavian artery steal syndrome) was also observed.

Facing the findings of carotid and subclavian involvement in a patient with constitutional symptoms, with evidence of high inflammatory activity and chest pain, a computed tomography angiography of the thoracic aorta was requested. Examination showed aneurysmatic dilation of the thoracic aorta with significant parietal thickening soon after the emergence of the left subclavian artery with a diameter of 24x28mm ( Figure 1 ).

**Figure 1:** - Computed tomography angiography. A) Saccular aneurysmatic dilation after the emergence of the left subclavian, partially thrombosed, measuring 4.2 cm in length and 2.4 x 2.8 cm in the largest diameters. B) Image obtained after 3D reconstruction.

Based on *American College of Rheumatology* 1990^1^diagnostic criteria, a hypothesis of giant-cell arthritis in extracranial form with aortic involvement was suggested. Corticotherapy was initiated with prednisone 60mg/day.

In the following days, the patient evolved with persistent hoarseness. Videolaryngoscopy was requested which showed paralysis of the left vocal cord ( Figure 2 ). Due to the anatomical correlation of the aneurysm with the left recurrent laryngeal nerve, the diagnosis of cardiovocal syndrome (or Ortner’s syndrome) was established.

**Figure 2:** - Image obtained by videolaryngoscopy shows asymmetry of the vocal cords, with signs of left vocal cord paralysis.

After corticotherapy, the patient obtained a significant improvement in constitutional symptoms and chest pain. Inflammatory markers after two weeks of treatment showed a significant drop (ESR: 20mm/1 hour and PCR: 1.0 g/L) and there was an improvement in anemia (Hb: 12.4 g/dl). In the 30-day follow-up, the patient remained asymptomatic with a slight improvement of hoarseness after starting phonoaudiology treatment

## Discussion

Giant-cell arteritis, even though it is a non-negligible condition in some situations, can be difficult to diagnose, especially when cranial symptoms such as temporal headache are absent. However, in the face of an appropriate epidemiological profile, associated with constitutional symptoms without clear explanation and evidence of involvement of large vessels, its diagnosis should be suggested.

Represents a condition that is more frequent in females in the proportion of 4:1. History of smoking, as in our case, increases 6-fold the risk of developing it.^5^The diagnostic criteria were established in 1990 by the American College of Rheumatology. Temporal artery biopsy showing an inflammatory granulomatous pattern is part of the criteria, but it lacks adequate sensitivity and can be avoided in the appropriate context in the presence of a suggestive clinical picture and with imaging studies compatible with the involvement of large vessels.^6^

The presence of a high erythrocyte sedimentation rate (usually over 55mm/1 hour) is a non-specific laboratory finding, but very frequently. The fast and effective response to corticotherapy strengthens the diagnostic possibility. Aortic involvement is not infrequent and early recognition, and treatment is essential to minimize acute and chronic complications.^7^

Ortner’s syndrome or cardiovocal syndrome, first described in 1897, is a rare situation characterized by compression of the recurrent laryngeal nerve by cardiovascular conditions leading to hoarseness, dysphagia and dysphonia.^8^Its association with giant cell arteritis is extremely rare and infrequent as an initial presentation likewise in our case.^9^

This case presents an unusual association whose recognition is fundamental for proper management and appropriate therapy in order to minimize adjacent complications of giant cell arteritis.
